# Supplementary material for: The Deubiquitinating Enzyme Inhibitor PR-619 Enhances the Cytotoxicity of Cisplatin via the Suppression of Anti-Apoptotic Bcl-2 Protein: In Vitro and In Vivo Study
Source: Cells. 2019 Oct 17;8(10):1268. doi: 10.3390/cells8101268 (PMC6830310; doi:10.3390/cells8101268)
Supplement: Supplementary file 1 [file cells-08-01268-s001.pdf]

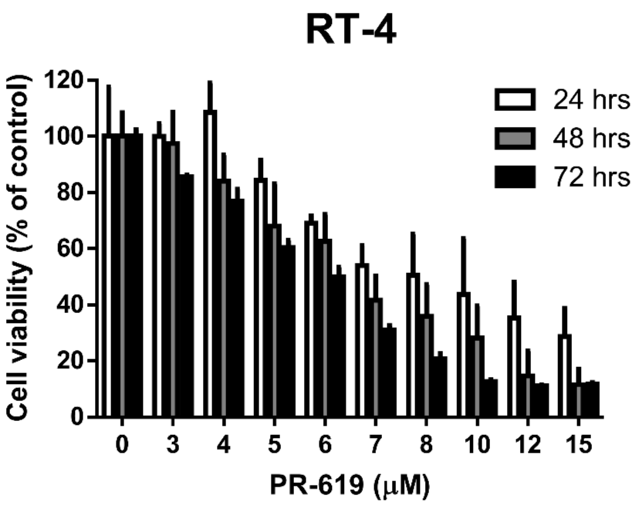

Figure S1. PR-619 induced cytotoxicity in low-grade UC RT-4 cells in a dose-dependent and time-dependent manner. RT-4 cells were treated with various concentrations of PR-619 (3–15 μM) for 24 h, 48 h, and 72 h, respectively. Cell viability was assessed using the MTT assay.

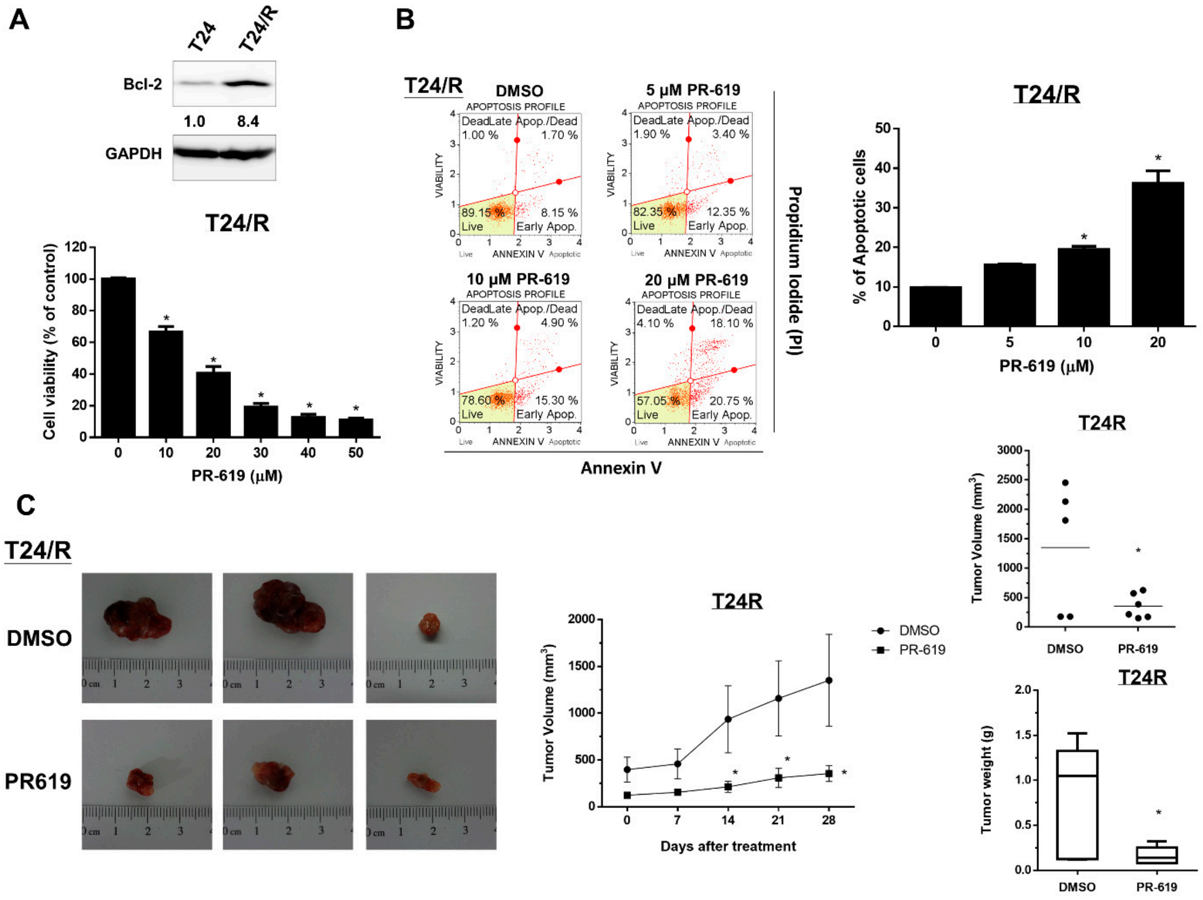

Figure S2. PR-619 effectively inhibited the cell viability and tumor growth of cisplatin-resistant UC (T24/R) *in vitro* and *in vivo*. (A) T24/R cells were treated

with various concentrations of PR-619 (10–50 μM) for 24 h. Cell viability was assessed using the MTT assay. (B) T24/R cells were treated with various concentrations of PR-619 (5, 10, and 15 μM) and DMSO for 24 h. Apoptotic cells were analyzed through FACS flow cytometry with propidium iodide and annexin V-FITC staining. The quantitative analyses of apoptosis are presented as the means ± SD.; *p < 0.05 compared with the control. (C) Nude mice bearing a cisplatin-resistant T24/R tumor xenograft over the bilateral flank area were treated with DMSO in normal saline (control, n=5) or PR-619 (10 mg/kg/day, i.p. (n=5)) for 4 weeks. Tumor images represent excised tumors from two groups. Weekly tumor volume for each group during the 4-week treatment. The tumor volume and weight in both groups after 4-week treatment are demonstrated. The data are presented as means ± standard error of the mean.

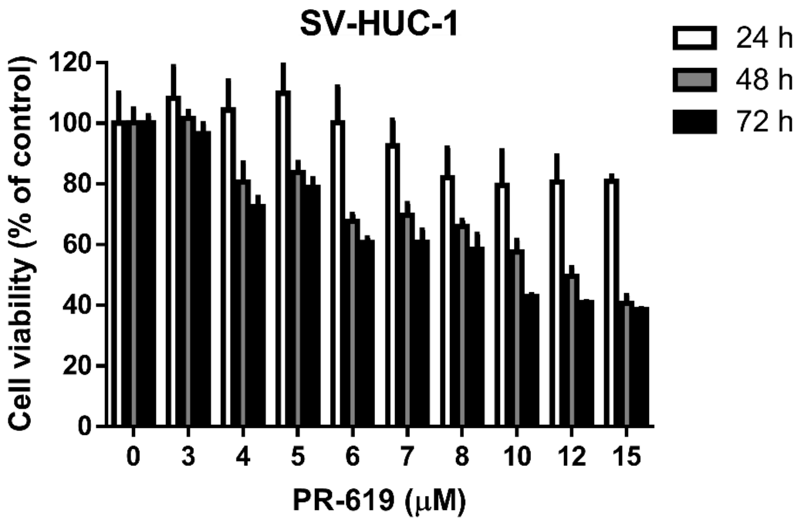

Figure S3. PR-619 elicited selective cytotoxic effects on UC cells compared to those on simian virus 40-transformed and immortalized human urothelial (SV-HUC-1) cells. SV-HUC-1 cells were treated with various concentrations of PR-619 (3–15 μM) for 24 h, 48 h and 72 h, respectively. Cell viability was assessed using the MTT assay.

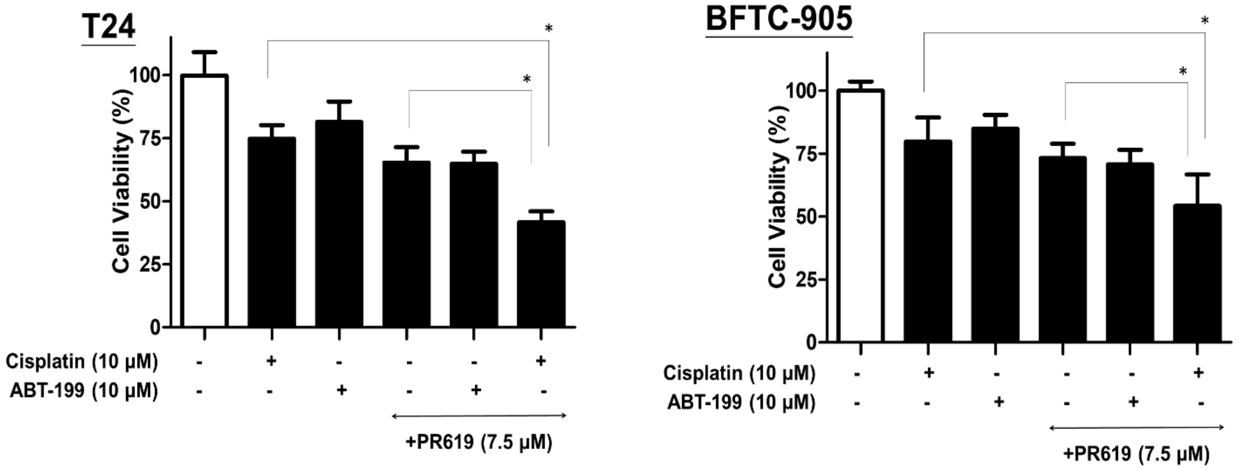

Figure S4. ABT-199, a Bcl-2 inhibitor elucidated limited impact on the cytotoxicity of PR-619. T24 and BFTC-905 cells were treated with various concentrations of ABT-199 (10 μM), cisplatin (10 μM), PR-619 (7.5 μM), alone or in combination for 24 h. Cell viability was assessed using MTT assay. Data were presented as the means ± SD. All results shown are representative of at least three independent experiments

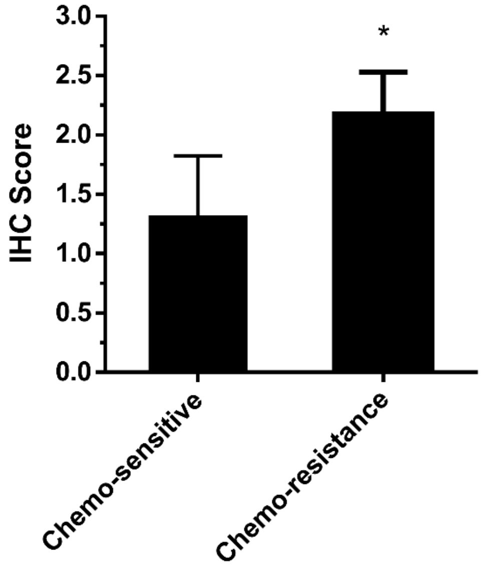

Figure S5. IHC score for measuring the immunoreactivity of Bcl-2 in bladder UC tissue samples from patients with metastatic UC with a chemo-resistant versus chemo-sensitive status. The method used to estimate the IHC score was described in the Materials and Methods section.
